# Presentation of ethical criteria during medical decision-making for critically ill patients: a mixed methods study

**DOI:** 10.1016/j.bjao.2022.100015

**Published:** 2022-06-09

**Authors:** Susanne Jöbges, Oliver Kumpf, Christiane S. Hartog, Claudia Spies, Ulrike Haase, Felix Balzer, Henning Krampe, Claudia Denke

**Affiliations:** 1 Department of Anaesthesiology and Operative Intensive Care Medicine (CCM/CVK), Charité Universitätsmedizin Berlin, Corporate Member of Freie Universität Berlin, Humboldt-Universität zu Berlin and Berlin Institute of Health, Berlin, Germany; 2 Department of Anaesthesiology, Surgical Intensive Care, Pain and Palliative Medicine, Hospital Dortmund, University Hospital Witten Herdecke, Dortmund, Germany; 3 Institute of Medical Informatics, Charité Universitätsmedizin Berlin, Corporate Member of Freie Universität Berlin, Humboldt-Universität zu Berlin, Berlin, Germany

**Keywords:** autonomy, clinical decision-making, design survey, ethics, patient will

## Abstract

**Background:**

Every medical decision is based on balancing medical knowledge, ethical considerations, and patient preferences. Previous surveys have mainly covered the ethical knowledge of medical staff. The aim of this study is to evaluate the feasibility of an innovative concept regarding how ethical criteria are applied to clinical decision-making during critical illness.

**Methods:**

An online survey including a short case vignette was carried out at a university hospital among physicians specialising in intensive care medicine in Germany. After free text responses regarding further required case information, the participants were asked to rank decision criteria during the course of the case vignette. A qualitative evaluation was performed by two independent investigators, based on a transcription into categories. This was followed by a quantitative analysis of ranked criteria.

**Results:**

Our analysis has shown that doctors are initially inclined to consider medical information when making treatment decisions. When complications occur, ethical values are more often included in the decision-making. The qualitative evaluation reveiled that the patient's will was consistently regarded as the leading criterion for decision-making. In the quantitative evaluation, patient's well-being, quality of life, and patient autonomy were rated as the most important decision criteria. Economic factors were ranked least important.

**Conclusion:**

A mixed methods approach is able to reflect the complexity of ethical reasoning within the medical decision-making process, suggesting the feasibility of this concept.

**Clinical trial registration:**

The study was registered under DRKS-ID: DKRS00011905 (April 2017).

There are critical situations every day in which good and sustainable decisions have to be made by the acting physician. Good clinical decision-making is based on evidence-based medicine and ethical reflection.^1^^,^^2^ Patient-centred, appropriate care involves individualised decisions, weighing the benefits and risks of the available treatment options in a timely way and considering the patient's wishes and values.^3^ Ethical principles can be helpful in defining the balance between what medical interventions are possible and what is appropriate.^4^ Regular reiteration of what a desirable outcome would be, and what would be permitted, can guide physicians in creating trust, aiding them to reach a shared decision with the patient and their relatives.^5^, ^6^, ^7^

In everyday clinical practice, however, complex decisions are influenced by personal, institutional, and socioeconomic factors, and are constrained by time. Therefore, we asked to what extent specific criteria affect decision-making in clinical situations. Today's medical ethical values are based on respect for autonomy and dignity, as reflected in declarations of human rights.^8^^,^^9^ Abandonment of paternalism is required to respect self-determination by the patient. Ethical values in medicine are present in the Hippocratic oath and the Declaration of Geneva.^10^ Childress and Beauchamp defined four fundamental ethical principles,^11^^,^^12^ which are compatible with different moral theories and linked to our moral convictions of everyday life:

- In the context of medical decisions, *autonomy* can be defined as recognition of individual life choices and self-determination. It includes the right to actively be part of shared decision-making as well as the right to refuse treatment methods. Informed consent serves as the basis for autonomy.^8^^,^^11^^,^^13^
- The principle of *justice* refers above all to a fair distribution of benefit and burden and is a very prominent professional value.^8^^,^^14^, ^15^, ^16^
- The principle of *non-maleficence* describes the central component of medical ethics, contained in reflection on the benefits/risks or harm of a therapeutic measure based on one of the promises from the Hippocratic oath, ‘first, do no harm’.^4^^,^^11^
- The principle of *beneficence* reflects ethical responsibility for the well-being of the patient.^2^^,^^17^ Well-being comprises at least two components. Firstly, the patient's individual view of his or her own well-being, and secondly an objective clinician's assessment of the health being promoted.^17^

Using an innovative design, we have tried to present these highly relevant ethical issues in medical decision-making. The question was: ‘Is it possible to depict the duality of medical and value-based decisions in the decision-making process?’

## Methods

### Qualitative approach and research paradigm

In order to provide a better understanding of the specific question of moral values in decision-making, we used an interpretivist qualitative approach in combination with a quantitative survey. Using qualitative and quantitative methods we wanted to reflect the specific points of view of the participants. The mixed methods approach makes it possible to elucidate more information than can be obtained in only quantitative research.^18^ We integrated qualitative data—individual comments—with the assessment of predefined medical ethics.

### Survey instrument

The innovative semi-structured online survey was designed by two intensive care physicians and two clinical psychologists with experience in intensive care, communication, and medical ethics. The survey consisted of two parts.

#### Part 1: semi-structured questionnaire (virtual case vignette)

**Fig 1:**
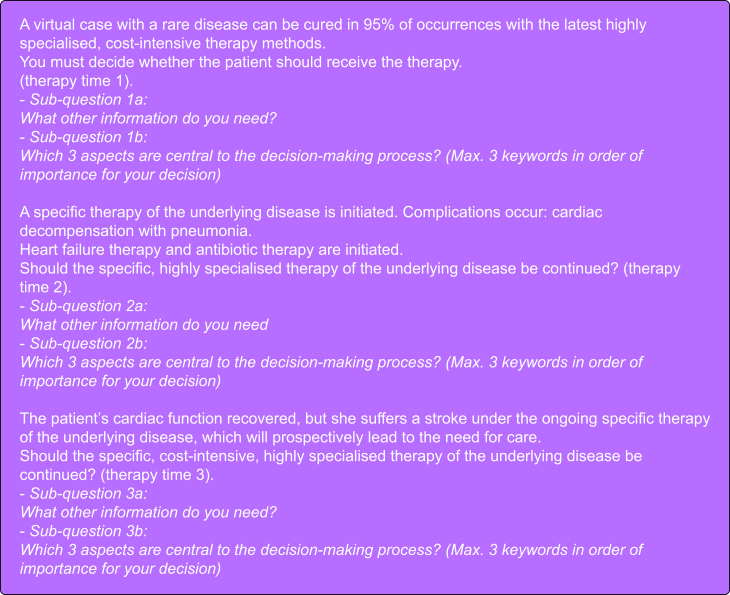
Semi-structured questionnaire (hypothetical scenario-virtual case vignette).

The semi-structured questionnaire (Fig. 1) contained a virtual case vignette with three defined timepoints. The first timepoint was the start of a cost-intensive therapy and recorded the approach and decisions made before initiating the intervention. The second timepoint dealt with ensuing complications, and at the third timepoint, the foreseeable outcome included a permanent need for long-term care: at these latter timepoints an increasingly poor prognosis was assumed.

At each virtual timepoint we asked three sub-questions with a maximum of three answers:

a) *What further information* was required for a decision on therapy.
b) *Which criteria* were considered for the respective therapy decision.
c) Please arrange the decision criteria in order of *importance*, with the most important being named first.

The answers could be freely formulated in each case.

#### Part 2: structured questionnaire

To objectively evaluate the criteria of a therapy decision, criteria were specified in a structured questionnaire. Predefined criteria included the four principles of Beauchamp and Childress,^11^ criteria of evidence, guidelines and standards to reflect evidence-based medicine,^1^^,^^19^ legal criteria defined as patient consent and the patient's autonomous decision-making,^20^ and economic considerations.^16^ The participants were asked to prioritise these criteria in the context of a therapy decision. The scale of evaluation ranged from ‘very unimportant=0’ to ‘highly relevant=5’.

### Sampling strategy

A voluntary, anonymous online survey was conducted among all physicians (*n*=195) from the Department of Anaesthesiology and Operative Intensive Care Medicine, Charité Universitätsmedizin Berlin, Campus Charité Mitte and Campus Virchow-Klinikum, over a period of 4 weeks in May 2017. In order to address all colleagues working in anaesthesiology who had previous intensive care experience, we sent an anonymised link to the entire medical staff of the department. The participants were informed that their decision to participate was voluntary and that data were to be used for research in an anonymised way. The study was approved by the institutional review boards (number EA4/137/16) and registered under DRKS-ID: DKRS00011905. The online survey was conducted using ‘Limesurvey Version 2’. We followed recommendations for qualitative studies by O'Brien and colleagues.^21^

### Data analysis

The qualitative evaluation of the semi-structured questionnaire (virtual case vignette) was carried out iteratively by two physicians with a medical background in ICU and further education in bioethics. The two physicians coded the answers to the open questions independently, shared their coding, discussed the different entries, found a consensus, and developed themes.^22^ In addition, we used Cohen's kappa to evaluate the degree of agreement. The categories ‘medical’, ‘legal’, and ‘ethical’ were formed *a priori*. During the evaluation it became apparent that the categories needed to be extended. The additional categories ‘patient will’ and ‘relatives and their involvement’ were created.

The final categories were defined as:

- Medical: statements about current clinical status, preliminary findings, previous illnesses, comorbidities, frailty, and side-effects of therapy.
- Ethical: statements about weighing up of benefit–risk ratio, harmfulness of treatment, quality of life, and cost.
- Patient will: answers about patient autonomy and patient will.
- Legal: answers regarding the capacity to give consent or the existence of a legal representative or advanced directive.
- Relatives and their involvement: answers with reference to relatives and their involvement in the decision-making process.

### Statistical analyses

The characteristics of the participants were evaluated descriptively. Categories developed during the qualitative analysis were presented according to frequencies per timepoint and answered questions. The mean scale values were calculated and arranged hierarchically for descriptive presentation. Numerical analysis of the predefined categories in the structured questionnaire was performed. The mean values (MV) were used to visualise a hierarchy of the given criteria.

## Results

We sent the questionnaire to 195 staff members, approximately 70 of whom were working most of the time in intensive care. Out of 34 responses received, 24 were evaluable (at least 21/39 items [nine open + 12 closed questions] answered). This resulted in an estimated response rate of 24/70 (34%). Of the 24 participants who provided evaluable data, 12 (50%) had practised medicine for 5–9 yr, and six (25%) for 10 yr or longer. Qualitative evaluation of the semi-structured part, the virtual case vignette, resulted in five categories (see Table 1). The Cohen's kappa as a measure of agreement had a median value of 0.80 with a range of 0.71–0.96.

### Synthesis and interpretation

#### Part 1: semi-structured questionnaire (virtual case vignette)

**Fig 2:**
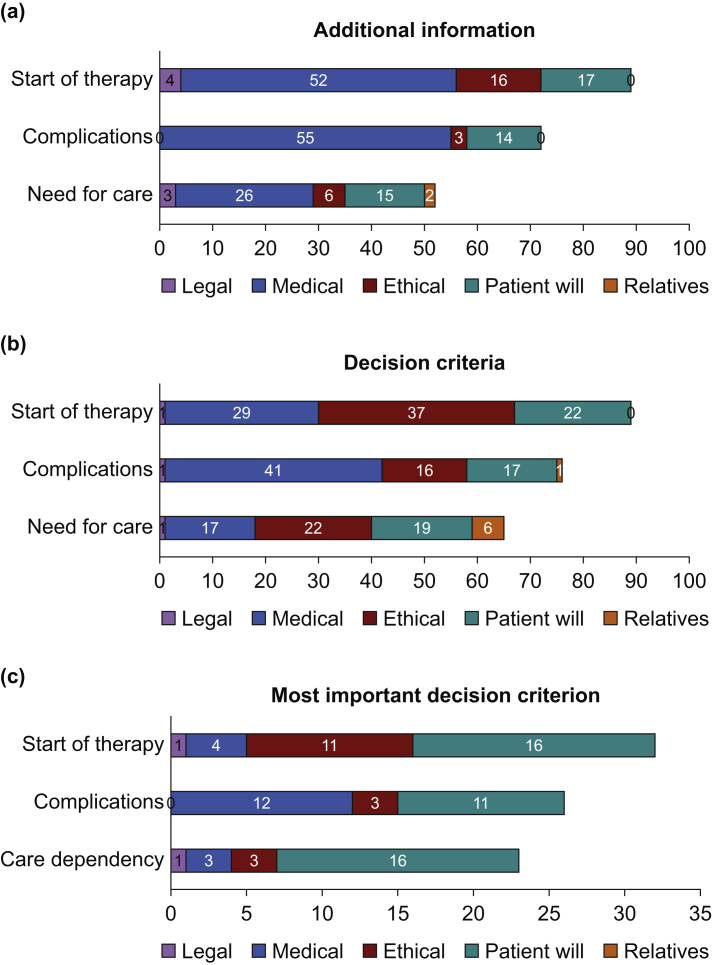
Criteria for decision-making based on a case vignette with increasingly poor progress over three therapy points. The following are meant by therapy times: 1: Basic therapy decision at the start of therapy. 2: Continuation despite complications, 3: Continuation despite foreseeable permanent need for care. The values in the bars represent absolute frequencies. The x-axis shows the number of answers.

*Required additional information*: early on, it became apparent that further medical information, such as previous diseases of the patient, frailty, or side-effects of the therapy was needed for the participants to reach a decision. With the onset of complications (therapy timepoints 2 and 3), additional ethical information was requested more often (e.g. harm as a result of further therapy) (Fig. 2a). Exemplary quotes for the five categories are presented in Table 1.

**Table 1:** Exemplary presentation of quotes in five categories. ID, identifier (anonymous answers were sampled by single consecutive numbers in the survey).

Category	Quote
Legal	ID 105: What is the patient's mental state? Has the patient's will been ascertained in advance or is there a written statement? (Advanced direction)? ID 123: How is the patient's ability to communicate + patient's involvement in decision-making? Clarification of healthcare proxies is necessary. (Power of attorney, advanced directives). ID 133: Is a living will in place? ID 136: What does the patient want? Does a living will exist? Does the patient have any relatives? The exact wording of the living will or the presumed will of the patient must be communicated. ID 141: Patient's ability to inform and consent.
Medical	ID 136: What are the patient's mobility, motivation, and other pre-existing conditions? Does the patient benefit from the therapy? ID49: What is the side-effect of the therapy on the medication used to treat the complications? ID105: Are there any other pre-existing conditions? A cancer? Other pre-existing conditions that could provoke a different therapeutic goal? ID 78: What is the long-term prognosis for the patient without continuation of the specific therapy?
Ethical	ID 29: Need to conduct a benefit/harm evaluation in conjunction with patient perceptions of individual/subjective quality of life. ID 31: What are the consequences of failure of the therapy? What is the achievable degree of improvement of the patient's condition considering the current state? ID 72: *nil nocere*: Does continuation of therapy further worsen the clinical condition? Feasibility of continued therapy? ID 81: Can an improvement in quality of life be expected despite the need for care? Benefit/risk assessment, therapy costs. ID 118: What is the benefit for the patient? Quality of life? The primary benefit of the therapy (cure) can no longer be expected. Costs must be considered. ID123: Interdisciplinary case discussion + reflection on resources to continue treatment even after discharge from hospital. ID 141: Discuss the achievability of the treatment goal in terms of functional outcome and quality of life. How important is the cure of the underlying disease compared with the need for care as a result of the stroke? Prioritisation of the patient's “values” (quality of life).
Patient will	ID 81: Does the patient wish to continue the therapy? How serious is the threat to the patient's general condition? ID 46: Patient's wish. Severity of need for care, degree of disability, degree of independence. ID96: What does the patient want? Patient's will? ID 141: Patient's wishes and values (social values), functional quality of life BEFORE the intervention.
Relatives	ID 123: Relatives of the patient must be involved. ID 81: Are there relatives/decision-making third parties who can be involved? ID 111: What is the possibility of further care after stroke in the home, by the relatives? ID 133: Discussion with relatives about presumed will of patient. ID 136: Clarification of the social environment (care possible, relatives, etc.)

*Decision criteria* which were considered in the therapy decision: ethical criteria (category ethics + patient will: 133 responses) outweighed the sum of medical criteria (87 responses) in the decision. With the occurrence of complications (sub-question 3), the perspective and opinion of the relatives was considered occasionally (Fig. 2b).

*Leading decision criterion*: the patient's will was more frequently mentioned in relation to ethical criteria, such as weighing up of benefit and risk, costs, or quality of life (Fig. 2c). Overall, the patient's will was for almost the entire process in all cases the leading and final decision criterion.

#### Part 2: structured questionnaire

**Fig 3:**
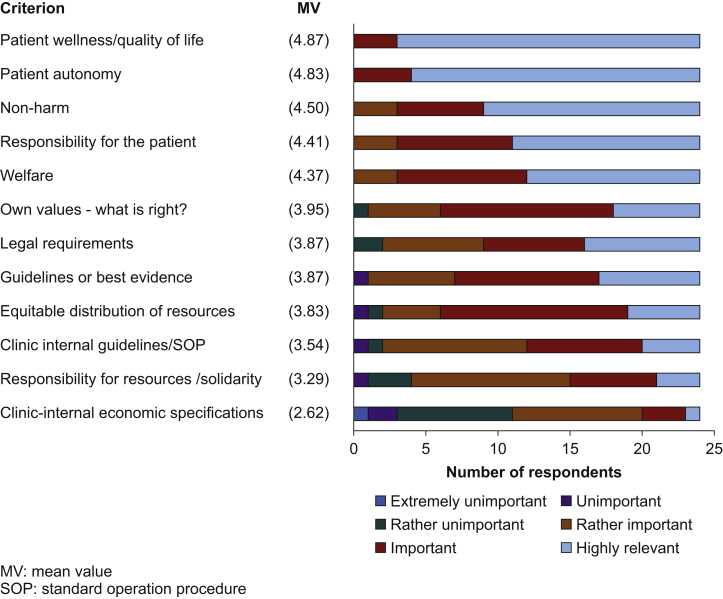
Criteria according to their importance for therapy decisions (*n*=24). MV, mean value; SOP, standard operating procedure.

The results of the prioritised criteria from the structured questionnaire are presented in Table 2 and Figure 3. Patient well-being/quality of life (MV 4.87) and patient autonomy (MV 4.83) were named as highly relevant. Other ethical principles, such as do no harm, responsibility for the patient in medical action and care, were also rated as important to highly relevant (MV between 4.5 and 4.37). Individual values of the physicians, however, and legal requirements were regarded as important (MV 3.95 and MV 3.87, respectively). The decision criteria guidelines/evidence (MV 3.87) and internal hospital guidelines such as standard operating procedures (SOPs) (MV 3.5) were rated as rather important to important by the participants. The topic justice, which included a fair distribution of resources (MV 3.83), responsibility for the community (MV 3.29), or economic guidelines (MV 2.65), was shown to be rather important.

**Table 2:** Ranking of criteria according to their importance for a therapy decision (*n*=24). Shown are the absolute frequencies of the answers. The categories were recorded numerically (‘very unimportant=0’ to ‘highly relevant=5’). The mean scale values were formed to create a hierarchy of the given criteria. ∗There was one answer missing in these categories.

	Extremely unimportant	Unimportant	Rather unimportant	Rather important	Important	Highly relevant	Mean value
Patient autonomy	—	—	—	—	4	20	4.83
Patient wellness/quality of life	—	—	—	—	3	21	4.87
Legal requirements	—	—	2	7	7	8	3.87

Equitable distribution of resources	—	1	1	4	13	5	3.83
Economic guidelines∗	1	1	8	9	3	1	2.65
Responsibility for the community of solidarity	—	1	3	11	6	3	3.29

Clinic-internal guidelines/SOP	—	1	1	10	8	4	3.54
Guidelines or evidence	—	1	—	6	10	7	3.87

Own values—what is right?	—	—	1	5	12	6	3.95
Welfare∗	—	—	—	3	9	11	4.35
Not to damage	—	—	—	3	6	15	4.5
Responsibility for the patient	—	—	—	3	8	13	4.41

## Discussion

**Fig 4:**
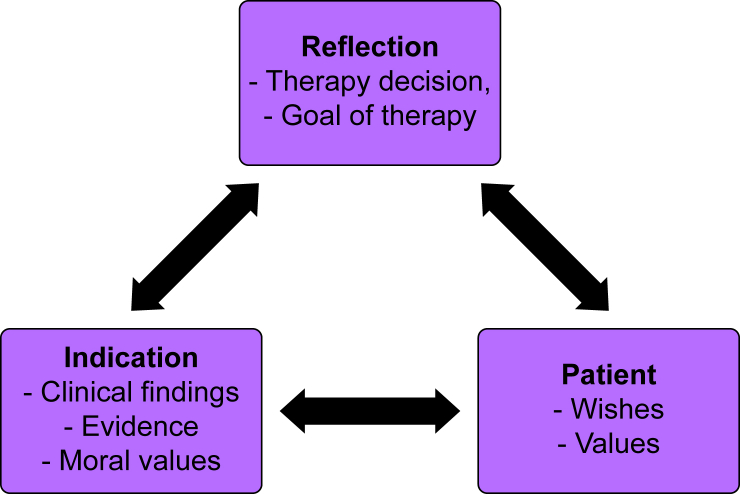
Design-model decision-making process.

**Fig 5:**
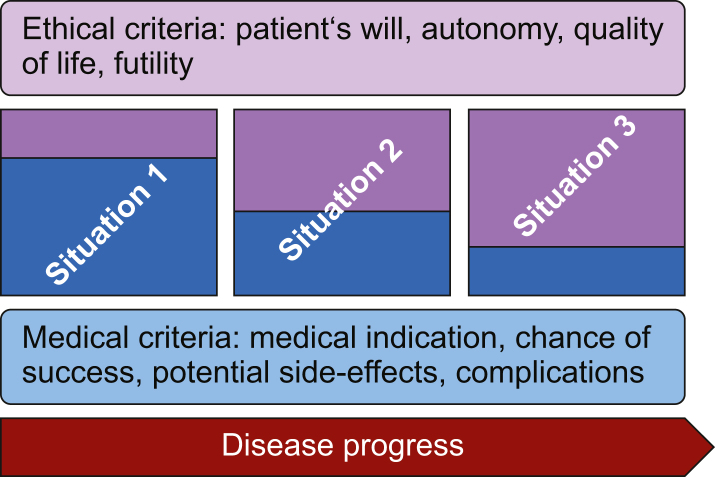
Shift of criteria in ethical decision-making depending on the disease progress. In the course of the disease process, criteria for ethical decision-making shift from medical criteria (denoted light blue) towards ethical ones (denoted light red) when success of treatment becomes more uncertain.

Medical decision-making is often multidimensional, associated with an uncertain outcome and influenced by the dynamics of a critical illness. The aim of the study was to evaluate a newly designed survey to describe ethical reasoning in the context of clinical decision-making in conjunction with critical illness. Using a design model (Fig. 4), our survey described medical and ethical considerations based on a virtual case vignette, and by ranking predefined criteria. The virtual case vignette proved that with the progressing clinical course, aspects of medical ethics became more prominent (Fig. 5). It was shown that, in the sum of categories, ethics and patient will always outweigh the sum of the medical criteria.

According to the Ethics Council of Germany, patient well-being includes ‘the enabling of self-determined care for patients, good quality of treatment, and the fair distribution of resources available for inpatient care’ (Ethics Council p. 132).^14^ The results of our study will be discussed based on this description.

### Self-determination

In a medical context, abandonment of paternalism is required to enable self-determination by the patient. In addition to the freedom to make decisions without coercion or manipulation, respect for self-determination also demands the support of self-determined decisions. The co-determination of a medical therapy (shared decision) within the framework of informed consent could be seen.^14^^,^^20^^,^^23^ In our study, patient will was mentioned as an outstanding ethical criterion in both parts of the questionnaire. Recognition of individual life choices and self-determination seems to play a major role in the medical staff's decisions.

The extent to which patient will/autonomy is considered by medical staff to be a legal or ethical criterion may vary. As an example, principles of autonomy/self-determination are part of the legal system, and human rights. The basic prerequisite for a medical intervention is the patient's consent and the examination of their ability to give consent.^20^ In the virtual case vignette, it was striking that only five participants addressed the issue of the ability to consent or the involvement of a legal representative. On average, legal requirements were rated as important. Thus, the patient's will seems to be accepted by doctors as the primary ethical criterion, but there may be uncertainties about the legal requirements. Corresponding to the concept of relational autonomy, therapeutic decisions for patients should be seen in the context of the social environment as well.^24^ The consideration of involving relatives in critical medical treatment decisions is also evident in our study. However, it does not play a role as a leading criterion.

### Care for the patient

Reflecting on the indication for any therapy ‘which is medically possible’, a review of the benefits and harms should be considered, with a focus on the long-term well-being of the patient.^8^^,^^25^ Reflection on the benefits and risks or harms of a therapeutic measure was shown by the participants in their increasing thought towards benefit/harm in the deteriorating virtual patient. In our study, patient well-being/quality of life was accorded the highest importance in the virtual case vignette. Quality of life encompasses both medical and ethical aspects and judgments must be adapted to the individual circumstances of each patient.^26^ The individual assessment and responsibility for the patient was made clear, in that the ethical principles of care and doing no harm scored highest in the questionnaire. The doctor's own values as a factor in an ethical reflection do not seem to play such an important role. The influence of religion and other value orientations as factors in an ethical reflection were not observed. Therapy decisions are subject to a special dynamic and individuality and the balancing of beneficence and harm is challenging.^27^

### Quality of treatment

Good quality treatment is demonstrated by applying interventions according to medical indications relevant to the individual patient.^4^^,^^19^ In the structured questionnaire, medical expertise was represented as part of good treatment quality through the question of guidelines and SOPs. It is striking that guidelines and SOPs seem to be subordinate to ethical values such as autonomy, patient well-being, benefit, and harm. One possible explanation is that available guidelines and SOPs do not yet sufficiently address an ethically differentiated evaluation.

### Distribution of resources

The principle of justice refers above all to distributive justice, and clear criteria for fair distribution within the framework of limited resources are necessary.^12^^,^^16^^,^^28^ This became apparent in the responses to the virtual case vignette, in which costs/resources were listed as decision criteria (‘latest highly specialised, cost-intensive therapy methods’). In the weighting of criteria, resources, responsibility for the community of solidarity, and internal economic guidelines were less important than other values. The fact that good clinical practice will favour the individual needs of a patient in the therapeutic approach might be a possible explanation. In this virtual case, justice can be thought of as: ‘The patient should receive everything that is necessary in terms of therapy’, which should be the guiding principle. Perhaps the respondents did not pay specific attention to the problem of limited resources. However, limited resources are evident in all healthcare systems and there is an ongoing debate about the allocation of resources in healthcare systems.^28^^,^^29^ It is necessary to include this reflection on resources and responsibility for the community.

A variety of decision theories are discussed in the context of intensive care treatment and practitioners.^27^ In addition to rational thinking, intuitive actions and emotional evaluations are incorporated into the decision-making process. Furthermore, external factors such as ICU environment and atmosphere impact on this process. In complex decision-making, information must be gathered and short-term and long-term outcomes weighed.^27^ The patient's condition may deteriorate acutely and new decisions become necessary. To narrow down the multifactorial process, this study focused on the case vignette and the required information related to the decision. It is impressive that our colleagues were focused on the patient's well-being during the decision-making process. Ethical criteria become most prominent when the goal of curative therapy cannot be achieved or severe complications occur. Better clinical education and reflection can help ensure that medical ethics criteria are present in every decision made regarding the individual patient.

Various forms of expression of medical ethical values present themselves in daily medical practice. According to a survey in England, 19 out of 27 universities use the Hippocratic oath or a variant (e.g. WMA Declaration of Geneva) as a sign of responsibility when entering the medical profession.^30^ In Germany, medical ethical values have been part of teaching since the licensing regulations came into force in 2003 to ensure that all prospective physicians in Germany have ‘comparable basic knowledge’.^31^ However, a definition of the basic contents of history, theory, ethics, and teaching, and their consolidation and promotion in training, is necessary.^32^

### Limitations

First, the low response rate among all physicians in the Department of Anaesthesiology was probably because the questionnaire focussed on those with critical care experience. In fact, the survey was completed satisfactorily by approximately one-third of the 70 physicians working fulltime in the ICU. Second, some of our conclusions could be subject to evaluation bias because it was not always possible to distinguish clearly between medical and ethical criteria in the categorisation of the free text answers. However, the high degree of agreement between the evaluating researchers reduces this risk. Third, the newly developed questionnaires have not yet been validated and so we cannot assert that all essential factors and processes have been included. Finally, the criteria included, which follow the medical-ethical principles of Beauchamp and Childress,^11^ are not without controversy. These principles have been criticised for not being directly applicable in acute medical emergencies as they require reflection. In acute situations, internalised moral values are used instead.^33^ It has also been argued that these ethical principles cannot be reflected in medical decision-making. Thus, medical-ethical principles are considered important, but there is no model for internalisation to integrate them within a decision-making process.^34^

The construction of a very clear case study was a great challenge in this study. The major reason for designing the vignette without giving additional information is that this information would have automatically influenced the participants' answers. Our major question asked what information the participants needed for their decision and which was most important. If we had provided more contextual information, we would have risked the participants focussing on missing information rather than which was most important. At first glance, designing a complex case by providing much clinical and social information might have been an option. However, providing all complex information would make the influence on the participants' answers even stronger, resulting again in biased answers. Obviously, the more complex a case is the more difficult it is to achieve qualitative results of high validity. In the complex decision-making process, a lot of information has to be collected and evaluated. Our study has shown that ethical values are present in this process.

## Conclusion

Our study shows that a mixed methods approach is able to reflect the complexity of ethical reasoning within medical decision-making, suggesting the feasibility of the concept. Using this survey, medical and ethical criteria in the decision-making process can be made comprehensible (Fig. 5). We found that physicians consider patient well-being and patient autonomy as leading ethical criteria for therapy decisions.

## Authors' contributions

Conception of the study: SJ, CS, UH.

Design of the survey: SJ.

Acquisition of data: SJ.

Analysis and interpretation of data: SJ, UH.

Drafting of the manuscript: SJ, CS, UH.

Revision of the manuscript: SJ, OK, CH, HK, CD.

Interpretation of data: OK, CH.

Creation of new software for the survey: FB.

Conception of the survey: HK, CD.

Approved the final manuscript: all authors.

## Declarations of interest

The authors declare that they have no conflicts of interest.

## Funding

The study was financed from funds of the clinic.

## Data availability

The datasets used, analysed, or both during the current study are available from the corresponding author on reasonable request.

## References

[1] Sackett D.L., Rosenberg W.M., Gray J.A., Haynes R.B., Richardson W.S. (1996). Evidence based medicine: what it is and what it isn’t. BMJ.

[2] Williams J.R. (2005).

[3] Coulter I., Herman P., Ryan G., Hilton L., Hays R.D. (2019). The challenge of determining appropriate care in the era of patient-centered care and rising health care costs. J Health Serv Res Policy.

[4] World Medical Association (2013). World Medical Association Declaration of Helsinki: ethical principles for medical research involving human subjects. JAMA.

[5] Merkl A. (2016). Ärztliches Ethos – gegenwärtige Herausforderungen ärztlichen Handelns. Z Med Ethik.

[6] Rosca A., Krones T., Biller-Andorno N. (2020). Shared decision making: patients have a right to be informed about possible treatment options and their risks and benefits. Swiss Med Wkly.

[7] Rabi D.M., Kunneman M., Montori V.M. (2020). When guidelines recommend shared decision-making. JAMA.

[8] Taylor R.M. (2013). Ethical principles and concepts in medicine. Handb Clin Neurol.

[9] Henry L.M., Rushton C., Beach M.C., Faden R. (2015). Respect and dignity: a conceptual model for patients in the intensive care unit. Narrat Inq Bioeth.

[10] Parsa-Parsi R. (2017). The revised Declaration of Geneva: a modern-day physician’s pledge. JAMA.

[11] Beauchamp T.L., Childress J.F. (2001).

[12] Beauchamp T.L., Rauprich O., Steger F. (2005). Prinzipienethik in der Biomedizin.

[13] Pasetti C. (2008). Bioethics and caregiving: a critical interaction in patient care today. G Ital Med Lav Ergon.

[14] The German Ethics Council (2016).

[15] Moyo M., Goodyear-Smith F.A., Weller J., Robb G., Shulruf B. (2016). Healthcare practitioners’ personal and professional values. Adv Health Sci Educ Theory Pract.

[16] World Health Organization Health equity. https://www.who.int/health-topics/health-equity#tab=tab_1.

[17] Bester J.C. (2020). Beneficence, interests, and wellbeing in medicine: what it means to provide benefit to patients. Am J Bioeth.

[18] Wisdom J., Creswell J.W. (2013). Mixed methods: integrating quantitative and qualitative data collection and analysis while studying patient-centered medical home models. https://www.ahrq.gov/sites/default/files/wysiwyg/ncepcr/tools/PCMH/mixed-methods.pdf.

[19] Fitch K., Bernstein S.J., Aguilar M.D. (2001).

[20] Robertsen A., Jöbges S., Sadovnikoff N., Michalsen A., Sadovnikoff N. (2020). Compelling ethical challenges in critical care and emergency medicine.

[21] O’Brien B.C., Harris I.B., Beckman T.J., Reed D.A., Cook D.A. (2014). Standards for reporting qualitative research: a synthesis of recommendations. Acad Med.

[22] Lewis J., McNaughton Nicholls C., Ormston R., Ritchie J. (2014).

[23] Tonelli M.R., Sullivan M.D. (2019). Person-centred shared decision making. J Eval Clin Pract.

[24] Grignoli N., Di Bernardo V., Malacrida R. (2018). New perspectives on substituted relational autonomy for shared decision-making in critical care. Crit Care.

[25] Jonsen A.R., Siegler M., Winslade W.J. (2015).

[26] Woopen C. (2014). [The significance of quality of life – an ethical approach]. Z Evid Fortbild Qual Gesundhwes.

[27] Alexis Ruiz A., Wyszyńska P.K., Laudanski K. (2019). Narrative review of decision-making processes in critical care. Anesth Analg.

[28] Persad G., Wertheimer A., Emanuel E.J. (2009). Principles for allocation of scarce medical interventions. Lancet.

[29] Emanuel E.J. (2000). Justice and managed care. Four principles for the just allocation of health care resources. Hastings Cent Rep.

[30] Green B. (2017). Use of the Hippocratic or other professional oaths in UK medical schools in 2017: practice, perception of benefit and principlism. BMC Res Notes.

[31] Federal Ministry of Justice and Consumer Protection. Approbationsordnung (2003). https://www.gesetze-im-internet.de/_appro_2002/.

[32] Schildmann J., Bruns F., Hess V., Vollmann J. (2017). “History, theory and ethics of medicine”: the last ten years. A survey of course content, methods and structural preconditions at twenty-nine German medical faculties. GMS J Med Educ.

[33] Christen M., Ineichen C., Tanner C. (2014). How “moral” are the principles of biomedical ethics? – A cross-domain evaluation of the common morality hypothesis. BMC Med Ethics.

[34] Page K. (2012). The four principles: can they be measured and do they predict ethical decision making?. BMC Med Ethics.

