# Supplementary material for: Presentation of ethical criteria during medical decision-making for critically ill patients: a mixed methods study
Source: BJA Open. 2022 Jun 9;2:100015. doi: 10.1016/j.bjao.2022.100015 (PMC10430832; doi:10.1016/j.bjao.2022.100015)
Supplement: Supplementary file 1 [file mmc1.docx]

**e-supplement:**

Supplementary Table 1b: Frequencies of response categories of the semi-structured questionnaire (case study)

| Table 1a | *"What further information do you require?"* | | | | |
| --- | --- | --- | --- | --- | --- |
|  | Legal | Medical | Ethical | Patient Will | Relatives |
| Therapy time 1 | 4 | 52 | 16 | 17 | 0 |
| Therapy time 2 | 0 | 55 | 3 | 14 | 0 |
| Therapy time 3 | 3 | 26 | 6 | 15 | 2 |

| Table 1b | "*Which 3 aspects are central to the decision-making process*?" | | | | |
| --- | --- | --- | --- | --- | --- |
|  | Legal | Medical | Ethical | Patient Will | Relatives |
| Therapy time 1 | 1 | 29 | 37 | 22 | 3 |
| Therapy time 2 | 1 | 41 | 16 | 17 | 1 |
| Therapy time 3 | 1 | 17 | 22 | 19 | 6 |

| Table 1c | "M**ost important** decision criterion" | | | | |
| --- | --- | --- | --- | --- | --- |
|  | Legal | Medical | Ethical | Patient Will | Relatives |
| Therapy time 1 | 1 | 4 | 11 | 16 | 0 |
| Therapy time 2 | 0 | 12 | 3 | 11 | 0 |
| Therapy time 3 | 1 | 3 | 3 | 16 | 0 |

Legend:

Therapy time 1 Initial decision to start therapy: "You must decide whether the patient should receive the therapy".

Therapy time 2 Continue despite complication: "Should the specific, highly specialized therapy of the underlying disease be continued? “

Therapy time 3 Continuation in case of need of care: "Should the specific, cost-intensive, highly specialised therapy of the underlying disease be continued
